# Stability in Reading Improvement After Home-Based Multi-Componential Training for Children with Developmental Dyslexia

**DOI:** 10.3390/brainsci16060636

**Published:** 2026-06-14

**Authors:** Elena Capelli, Sara Mascheretti, Enrica Rosso, Patrizia Bernasconi, Renato Borgatti, Serena Lecce, Alessandra Piccolini, Simonetta Cardinali, Cristiano Termine, Laura Farinotti

**Affiliations:** 1 Department of Brain and Behavioural Sciences, University of Pavia, 27100 Pavia, Italy; renato.borgatti@unipv.it (R.B.); serena.lecce@unipv.it (S.L.); 2 Child Psychopathology Unit, Scientific Institute, IRCCS Eugenio Medea, 23842 Bosisio Parini, Italy; 3 IRCCS Mondino Foundation, 27100 Pavia, Italy; enrica.rosso@mondino.it (E.R.); patrizia.bernasconi@mondino.it (P.B.); laura.farinotti@mondino.it (L.F.); 4 Dipartimento Salute Mentale e Dipendenze, ASST Pavia, 27100 Pavia, Italy; alessandra_piccolini@asst-pavia.it (A.P.); simonetta_cardinali@asst-pavia.it (S.C.); 5 Child Neuropsychiatry Unit, Department of Medicine and Technological Innovation, University of Insubria, 21100 Varese, Italy; cristiano.termine@uninsubria.it

**Keywords:** developmental dyslexia, home-based training, age, follow-up, stability

## Abstract

**Background:** RIDInet-Reading Trainer 2 (RT-2) is a web-platform for the remote treatment of developmental dyslexia (DD) which has been shown to improve reading performance. However, no previous studies have investigated stability in reading improvement after RT-2 training and the influence of a previous diagnosis of developmental language disorder (DLD) and of participants’ age on stability. **Objectives:** In a sample of 52 Italian-speaking children with DD who participated in a 3-month home-based treatment with RT-2, we aimed (1) to assess the stability in reading improvement after RT-2 training at a 3-month follow-up and the potential moderating role of DLD and age; and (2) to evaluate the impact of RT-2 training in reading comprehension. **Results:** By implementing linear mixed model analysis, our findings confirmed reading improvement after RT-2 training in word and text reading in DD. Moreover, we observed an overall stability in single-word and text reading speed performances after three months, regardless of the diagnosis of DLD and the age of the participants. Conversely, accuracy showed an overall stability for single-word reading, while it was significantly stable only in the younger participants in text reading. The improvement was educationally relevant as it impacted reading comprehension. **Conclusions:** The current study supports the use of remotely delivered DD interventions among school-aged children.

## 1. Introduction

Reading requires the integration of multiple cognitive and sensory systems, supported by extensive neural circuits, to transform arbitrary sequences of visual symbols into meaningful sounds, words, sentences, and paragraphs [1]. Developmental dyslexia (DD), a complex neurodevelopmental disorder, affects approximately 7% of school-age children across various linguistic and cultural backgrounds. It primarily impairs the acquisition and effective use of reading skills in both academic and daily life contexts. Despite intact neurological and sensory functions, adequate educational opportunities, and average intelligence, individuals with DD experience persistent challenges in reading and literacy-related tasks [2], often resulting in secondary functional difficulties and negative psychosocial outcomes [3,4].

Following the mobility restrictions imposed due to the COVID-19 pandemic, there has been a significant increase in the focus on telemedicine and the delivery of healthcare services through digital platforms. The World Health Organization [5] defines telemedicine as the provision of healthcare services via technology to generate beneficial health outcomes for individuals [6]. Technologies such as computers, tablets, and smartphones—widely used in daily life across the globe—have become essential components of telemedicine [7]. Their application spans across various medical disciplines and age groups, underscoring their growing importance in modern healthcare [8]. While these approaches have generally demonstrated effectiveness, conclusive evidence regarding their efficacy remains limited, particularly in the context of neurodevelopmental disorders [9]. Preliminary studies suggest that remote interventions can enhance treatment efficiency by optimizing the duration and flexibility of therapy while maintaining feasibility and patient engagement [10,11,12]. Furthermore, remote interventions may contribute to reducing waiting lists and rehabilitation costs while mitigating the psychological, social, and educational consequences associated with delayed treatment [13,14,15].

Until now, various remediation treatments targeting one specific reading-related sensory and cognitive component have been implemented, e.g., phonological skills [16,17,18], auditory processing [19,20,21,22], rapid automatized naming (RAN) [12,23], executive functions [24,25,26], visual attention [27,28,29,30,31]. Phonology-based training, including letter–speech sound training and phonics instruction, has been shown to lead to significant improvements in reading fluency, particularly in the speed and automatization of grapheme–phoneme associations [16,17], supporting the hypothesis that deficits in these processes contribute to dyslexic reading difficulties. Consistently, phonics-based interventions yielded stronger improvements in non-word reading accuracy and fluency [16,17], while phonological awareness training showed indirect positive effects on reading outcomes among children with below-average language abilities [18], suggesting particular benefits for individuals at linguistic risk for reading difficulties. Recent studies have shown that visual-attention training (including tachistoscopic stimulation and action video games) may positively influence reading abilities in children with DD by enhancing attentional and decoding processes. Specifically, visual-attention training, together with enhanced attentional control abilities, was associated with significant improvements in decoding, particularly in pseudo-word reading speed and accuracy, accompanied by significant gains in text reading accuracy, reading rate, and reading comprehension [27,28,29]. These findings support the hypothesis that strengthening visual–spatial attention and temporal processing may facilitate efficient decoding and reinforce the visuomotor processes that contribute to fluent reading, supporting the role of these interventions in fostering automatization processes involved in lexical access [30,31].

Notably, the recent literature has introduced novel intervention tools that integrate multiple cognitive domains that appear to yield greater improvements in reading skills; however, findings on this topic are still scarce. For example, combining lexical and attentional training [32,33,34] or integrating executive function training with phonological training [35] has resulted in significant enhancements in reading fluency and accuracy in both clinical and general population samples. In recent years, emerging evidence has supported the efficacy of remotely administered remediation treatments, particularly in Italian-speaking populations. Notably, Lorusso and colleagues [36,37] have demonstrated promising outcomes in the remote administration of combined lexical and attentional training for DD intervention. Although evidence is still sparse for what concerns the long-term stability of these effects and educational relevance, initial findings support the stable effects and academic outcomes of multi-component training. Multi-component training tapping visuo-attention and lexical access seem to lead to stable increases in global reading speed and accuracy after six months [36,37]. Similarly, the improvement in reading speed and accuracy, as well as in attentional control and planning observed after a multi-component training of executive functions and phonological skills were largely maintained at a 6-month follow-up and positively affected school grades [35].

The fee-based RIDInet online platform [38] provides intensive, self-adaptive training activities that are remotely monitored by clinicians. These activities have been validated for home-based treatment, demonstrating good feasibility and efficacy [39,40]. Among them, Reading Trainer 2 (RT-2) [41] is specifically designed to enhance reading decoding and speed. RT-2 includes multi-componential features that support sublexical and lexical processes [42,43], as well as the visual and visuo-spatial aspects of reading [44,45]. During training, the child reads aloud narrative texts displayed on a computer screen at a pre-set speed (syllables per second), which progressively increases based on reading accuracy (measured by the number of errors). The clinician can customize the reading units (letters, syllables, morphemes, or words) and their presentation—either displayed individually on the screen or highlighted within the text according to the pre-set speed. Reading accuracy is recorded by an adult assisting the child, who presses the “Enter” key whenever a decoding error occurs. The training includes narrative texts with varying levels of difficulty, based on text length, word complexity, and frequency. Empirical studies have demonstrated the efficacy of RT-2 in improving reading performance in children with DD [10,15,39,40]. Specifically, previous studies have consistently reported significant improvements in reading decoding following intervention in individuals with dyslexia, particularly in reading speed and accuracy for text, words, and pseudo-words [12,15,39,40]. Notably, these gains were greater than those expected from spontaneous developmental progression alone [40] and appeared independent of functional neuropsychological profile or history of oral language delay [15]. While treatment effects were specific to decoding and did not extend to reading comprehension or spelling skills, improvements were also observed in cognitive functions considered precursors of reading ability, including rapid lexical access, phonological processing, and visual attention [15].

### Purpose of the Present Study

Taken together, these studies show that RT-2 can effectively increase reading skills after only a few months of home-based training. However, to the best of our knowledge, no previous studies have investigated the stability in reading improvement after RT-2 training. Long-term follow-ups of reading interventions are scarce and further investigation on this topic is crucial. On one hand, follow-ups allow us to test the effect of training on the automatization of reading; on the other hand, as DD has a relevant impact on everyday life, information on improvement retention is crucial for patients’ wellbeing and care planning. Furthermore, previous findings showed that having a diagnosis of developmental language disorder (DLD) has a substantial and enduring impact on reading development and is closely linked to the risk of DD [46,47,48,49,50,51,52,53,54]. Research on preschool children further indicates that comorbid language impairment exacerbates early deficits in phonological processing, verbal memory, and emergent literacy skills, which are foundational for successful decoding and later reading fluency, thereby increasing vulnerability to DD [54]. Although structured treatments can improve decoding skills, comorbid language impairments could constrain the magnitude, rate, and generalization of these gains [55,56,57]. Nevertheless, much of the existing literature has focused on comparisons between DD and DLD as independent groups, without systematically investigating children who meet the criteria for both. This highlights an important direction for future research, particularly given the potential complexity of their reading profiles [56]. Accordingly, empirically investigating the role of DLD comorbidity on the long-lasting effects of reading interventions has crucial implications for clinical practice [58]. Gaining a better understanding of their differentiated profiles is essential for the development of accurate diagnostic tools and tailored intervention approaches. In addition, evidence about age’s effect on the stability of the reading skills’ improvement is scarce. There is a general agreement that treatment in younger children with DD is preferred to maximize educational potential. However, as reading is a progressive developmental skill that builds upon prior knowledge to master increasingly complex abilities [59], it is important to assess potential differences across different stages of reading acquisition [36]. In a sample of 52 Italian-speaking children with DD who underwent RT-2 and a neuropsychological assessment of reading skills before, after, and at a 3-month follow-up, we aimed: (1) to assess whether reading improvement after RT-2 training was maintained after a 3-month follow-up. Moreover, we tested whether this potential stable effect can be moderated by the presence of DLD (AIM 1.1) and by possible age-related differences tapping different stages of reading acquisition (initial/intermediate and consolidated; AIM 1.2); and, (2) to evaluate the impact of RT-2 training in an educationally relevant task (i.e., reading comprehension).

## 2. Materials and Methods

### 2.1. Sample

The overall sample comprised 52 children (25 males) aged between 7 and 13 years (mean age = 9.08, SD = 1.42). Children were recruited at IRCCS Mondino Foundation (Pavia, Italy). They were included in the study if: (a) they received a clinical diagnosis of developmental dyslexia (ICD-10 codes: F81.0 or F81.3) and performed below −1.5 SD in at least two measures of reading parameters (accuracy and/or speed in single words, pseudo-words, and text reading) at the pre-intervention assessment; (b) had total IQ score above 70 on the Wechsler Intelligence Scale for Children—Fourth Edition [60]; (c) were Italian native speakers; and, (d) did not present comorbidity with other neurological conditions and/or sensory impairments. Among these participants, 32 subjects (61.5%) had a previous clinical diagnosis of DLD. Socio-demographic (i.e., maternal and paternal education, expressed as years of schooling) and clinical description of the sample at first assessment can be found in Table 1 (post and follow-up z-scores can be found in Supplementary Materials S1). Overall, at first assessment, 33 subjects (63.5%) experienced difficulties in reading comprehension (with below grade average performance). Twenty-five children (48%) were no longer available at the follow-up (T2) due to the COVID-19 pandemic or family reasons not related to the treatment (e.g., moving, previous contacts no longer active). However, children who were not available at T2 did not significantly differ in socio-demographic and clinical measures from children who underwent the follow-up (Supplementary Materials S1). The study was approved by Lombardy Region—PROGETTO REGIONALE G044 “Disturbo specifico dell’apprendimento: progetto di diagnosi, riabilitazione, presa in carico e formazione” (approval on 1 May 2015) and conducted in accordance with the ethical standards of the 1964 Helsinki Declaration and its later amendments or comparable ethical standards. All parents were informed of the study goals and procedures, and their written informed consent was obtained before the beginning of the study.

### 2.2. Design

A test–training–retest follow-up experimental design was carried out. All children were tested individually at three time-points: pre-intervention (T0), post-intervention (T1), and after three months of the end of the treatment (T2). Children took part in a remote intervention program, using the RIDInet RT-2 software for at least 36 training sessions over a maximum of 12 weeks, with a minimum average of three accesses per week as recorded by the RIDInet RT-2 software (mean = 5.52, SD = 1.47, minimum = 3.00, maximum = 7.00). The training did not have a fixed working schedule, so as to adapt to the child’s rhythms and attention capacity, but the children were encouraged to work at least 4–5 days per week in sessions of 15–20 min. The program included one pre-intervention meeting in order to teach the families how to use the RIDInet RT-2 software and for programming the first parameters.

### 2.3. Neuropsychological Assessment

All children underwent the following neuropsychological battery at T0, T1 and T2:

1. Single-word/pseudo-word reading from *DDE-2: Batteria per la Valutazione della Dislessia e Disortografia Evolutiva-2* [61]. The task assesses speed and accuracy (number of errors) in reading single-word lists (4 lists of 24 words) and single pseudo-word lists (3 lists of 16 pseudo-words) and provides grade norms from the second grade of primary school to the last grade of middle school. Reliability was >0.70 for speed (both single words and pseudo-words) and >0.55 for accuracy (both single words and pseudo-words); concurrent validity was above >0.70 [61]. Standardized z-scores were used in the analyses.
2. Text reading from *Prove di rapidità e correttezza nella lettura del gruppo MT* [62]. The task assesses reading skills (speed and accuracy, number of errors) for meaningful texts. Texts increase in complexity with grade level, and norms are provided for each text. Test–retest reliability was >0.80 and >0.70 for speed and accuracy, respectively [62]. Standardized z-scores were used in the analyses.
3. Text reading comprehension from *Prove di rapidità e correttezza nella lettura del gruppo MT* [62]. The task assesses written reading comprehension for meaningful texts with multiple choice questions (number of correct answers). Texts increase in complexity with grade level, and norms are provided for each text. Test’s norms provided a 4-level categorical performance score and not a continuous one (e.g., z-score). In order to assess impact in the academic domain, the final score was re-coded as a dichotomous variable according to the individual performance, i.e., below-grade-expected score *versus* at-grade level, because the aim was to assess whether there was a clinically relevant effect after the RT-2 training (shifting from a deficit to acceptable comprehension for grade). Test–retest reliability was >0.50 [62].

Neuropsychological assessment was done in person and administered by a speech therapist in a quiet hospital room.

### 2.4. Plan of Analyses

*A priori* power analysis was conducted using the G*Power software, Version 3.1.9.7 [63], to estimate sample size. As a model, we selected “ANOVA: Repeated measures, within–between interaction”; α was set at 0.05, f = 0.20, 1-beta = 0.80. Under these assumptions, the minimal sample size was equal to 50.

To reach our aims, we followed a two-step procedure. To preliminary test the RT-2 training’s effectiveness (i.e., reading improvement after RT-2 training), we ran a linear mixed model analysis with each neuropsychological measure as dependent variable, time-point (T0—pre; T1—post) as a fixed effect, and subjects as random effect, e.g., Word reading speed (z) ~ 1 + Time-point + (1|ID).

Stability in reading improvement after RT-2 training (AIM 1) was inspected only using neuropsychological measures for which we found a significant improvement after RT-2 in the preliminary analyses (i.e., single words and text reading, both speed and accuracy). For this purpose, we ran a linear mixed model analysis with each neuropsychological measure (z-scores) for which we found a significant improvement after RT-2 training as the dependent variable, performance at different time-points (T0—pre; T1—post; T2—follow-up) as the fixed effect, and subjects as the random effect. For all the estimated models, we split the participants in two groups according to the presence or absence of a diagnosis of DLD (yes—present; no—absent) (AIM 1.1), and the different age ranges (median split: younger < 9 years of age n = 25, and older ≥ 9 years of age n = 27) to assess potential effects of age on reading improvement (AIM 1.2), e.g., Word reading speed (z) ~ 1 + DLD/Age group * Time-point + (1|ID). The choice of entering age as a categorical factor using a median split was motivated by the non-normal distribution of age (skewness = 1.13, kurtosis = 1.45; Shapiro–Wilk W = 0.92, *p* = 0.001) and by theoretical considerations. Specifically, the split approximates the transition described by Chall [59] from a “learning-to-read” stage to a “reading-to-learn” stage, enabling a theoretically meaningful comparison between developmental phases. Interaction terms between intervention and groups were also estimated *via* F-test, and post hoc analyses were conducted (where needed) assessing paired comparisons (marginal mean differences *t*-test).

For AIM 2 (i.e., the impact of RT-2 training in reading comprehension), two paired-sample chi-square tests were computed to evaluate changes in reading comprehension levels (i.e., below-grade-expected score vs. at-grade level) between T0 and T1 and between T1 and T2.

Analyses were conducted with Jamovi software version 2.6.24 [64]; setting alpha < 0.05.

## 3. Results

### 3.1. Preliminary Analyses—Reading Improvement After RT-2 Training

Concerning single-word reading, a significant improvement emerged after the intervention in both reading speed (F(1, 51.0) = 13.60; *p* < 0.001) and reading accuracy (F(1, 51.0) = 12.60; *p* < 0.001). For single pseudo-words reading, neither reading speed (F(1, 50.7) = 2.33; *p* = 0.133) nor reading accuracy (F(1, 51.0) = 1.73; *p* = 0.194) reached significance, indicating no immediate effect of the intervention on pseudo-word reading. Finally, for text reading, significant improvements were observed both in reading speed (F(1, 51.0) = 24.00; *p* < 0.001) and reading accuracy (F(1, 51.0) = 8.97; *p* = 0.004) from T0 to T1 (see Figure S1 in Supplementary Materials S2).

### 3.2. AIM 1—Stability in Reading Improvement After RT-2 Training

For single-word reading speed, the effect of intervention including follow-up was significant (F(2, 77.9) = 4.55; *p* = 0.014) even if the overall stability of the intervention’s improvement at T2 did not reach conventional statistical significance (T2 vs. T0: t(80.4) = −1.84, *p* = 0.070; T2 vs. T1: t(80.4) = 0.41, *p* = 0.682). In the case of single-word reading accuracy, a significant intervention effect was observed (F(2, 77.0) = 7.85; *p* < 0.001) with overall stability of gains also observed at follow-up (T2 vs. T0: t(79.1) = −2.75, *p* = 0.007; T2 vs. T1: t(79.1) = 0.08, *p* = 0.936) (see Figure S2 in Supplementary Materials S2). Results did not change after controlling for Global IQ (see Supplementary Materials S2).

### 3.3. AIM 1.1—Stability in Reading Improvement After RT-2 Training and DLD Comorbidity

**Figure 1:**
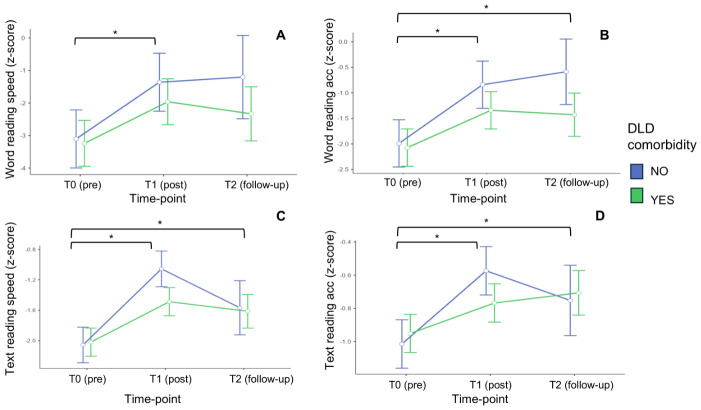
Pre-post training (3 months) and follow-up (after 3 months) performance (mean and standard error) in word reading speed (**A**), accuracy (**B**), text reading speed (**C**), and accuracy (**D**) for both DLD sub-groups (yes *versus* no comorbidity). * *p* < 0.05.

Concerning single-word reading speed, a significant main effect of intervention emerged (F(2, 76.5) = 4.70; *p* = 0.012), even if the overall stability of the intervention’s improvement at T2 did not reach conventional statistical significance (T2 vs. T0: t(79.6) = −1.93, *p* = 0.057; T2 vs. T1: t(79.6) = 0.15, *p* = 0.884). No significant main effect in the DLD group was found (F(1, 54.7) = 0.37; *p* = 0.546), nor a significant intervention ×DLD interaction (F(2, 76.5) = 0.26; *p* = 0.775) (Figure 1A). Coherently, the model explained only 3.30% of the variance through fixed effects (marginal R^2^ = 0.033), while the full model including random effects explained 59.70% of the variance (conditional R^2^ = 0.597). Similarly, for single-word reading accuracy, a significant main effect of intervention emerged (F(2, 75.5) = 8.57; *p* < 0.001) with significant improvement stability at T2 (T2 vs. T0: t(78.0) = −2.93, *p* = 0.005; T2 vs. T1: t(78.0) = −0.24, *p* = 0.812). Neither the main effect of the DLD group (F(1, 53.5) = 0.78; *p* = 0.382) nor the intervention × DLD group interaction reached statistical significance (F(2, 75.5) = 0.71; *p* = 0.497) for single-word reading accuracy (Figure 1B). The model explained 5.30% of the variance through fixed effects (marginal R^2^ = 0.053), while the full model explained 66.20% of the variance (conditional R^2^ = 0.662).

With regard to text reading speed, the analysis revealed a significant effect of intervention (F(2, 78.7) = 12.31; *p* < 0.001). Paired comparison post hoc analyses revealed an overall stability in improvement after the intervention at T2 (T2 vs. T0: t(82.9) = −2.10, *p* = 0.039; T2 vs. T1: t(82.9) = 1.49, *p* = 0.140). No significant main effect of the DLD group was observed (F(1, 55.6) = 0.32; *p* = 0.573), and the intervention × DLD group interaction was not significant (F(2, 78.7) = 1.22; *p* = 0.301) (Figure 1C). The model explained 9.90% of the variance through fixed effects (marginal R^2^ = 0.099), while the full model explained 51.70% of the variance (conditional R^2^ = 0.517). As for text reading accuracy, results indicated a significant main effect of intervention (F(2, 80.8) = 6.73; *p* = 0.002) with significant improvement stability after the intervention, also at T2 (T2 vs. T0: t(84.0) = −2.08, *p* = 0.040; T2 vs. T1: t(84.0) = 0.48, *p* = 0.630). The main DLD group (F(1, 58.1) = 0.03; *p* = 0.865), and intervention × DLD group interaction (F(2, 80.8) = 1.20; *p* = 0.307) effects were not statistically significant (Figure 1D). The model explained 5.00% of the variance through fixed effects (marginal R^2^ = 0.050), while the full model explained 57.70% of the variance (conditional R^2^ = 0.577).

### 3.4. AIM 1.2—Stability in Reading Improvement After RT-2 Training and Age-Related Differences

**Figure 2:**
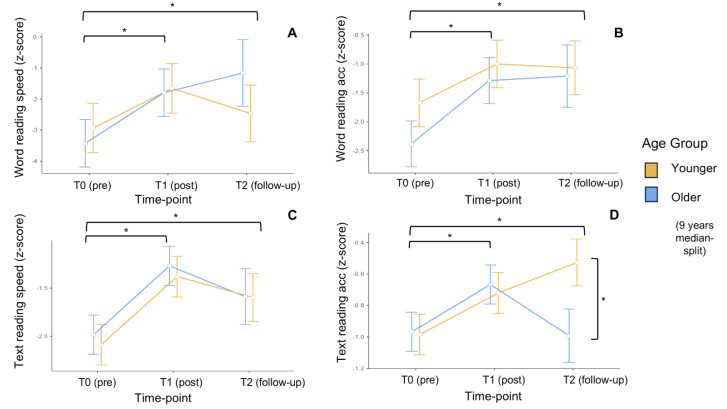
Pre-post training (3 months) and follow-up (after 3 months) performance (mean and standard error) in word reading speed (**A**), accuracy (**B**), text reading speed (**C**), and accuracy (**D**) for both age sub-groups (younger *versus* older than 9 years). * *p* < 0.05.

Looking at single-word reading speed, a significant main effect of time emerged (*F*(2, 76) = 4.69; *p* = 0.012). Paired comparison post hoc analyses revealed an overall stability of the intervention’s improvement at T2 (T2 *vs.* T0: t(74) = −2.04, *p* = 0.005; T2 *vs.* T1: t(80) = 0.13, *p* = 0.896). The model explained 3.52% of the variance through fixed effects (marginal R^2^ = 0.035), while the full model including random effects explained 59.70% of the variance (conditional R^2^ = 0.597). Concerning single-word reading accuracy, the main effect of time was significant (*F*(2, 75) = 7.70; *p* < 0.001), and paired comparison post hoc analyses revealed an overall stability in improvement after RT-2 training also at T2 (T2 *vs.* T0: t(74) = −3.63, *p* < 0.001; T2 *vs.* T1: t(79) = 0.02, *p* = 0.987). No age group nor age group×intervention interaction effects on single-word reading speed and accuracy emerged (*p*s > 0.05) (Figure 2A,B). The model explained 5.71% of the variance through fixed effects (marginal R^2^ = 0.057), while the full model explained 65.78% of the variance (conditional R^2^ = 0.658).

Referring to text reading speed, a significant main effect of time was highlighted (*F*(2, 77) = 10.96; *p* < 0.001). Paired comparison post hoc analysis showed overall stability in improvement after RT-2 training at T2. The difference between T0 and T2 was significant (*t*(75) = −4.66; *p* < 0.001), whereas the T1 and T2 difference was not (*t*(83) = 1.36; *p* = 0.176). No age group nor age group*intervention interaction effects were found (ps > 0.05) (Figure 2C). The model explained 8.58% of the variance through fixed effects (marginal R^2^ = 0.086), while the full model explained 50.38% of the variance (conditional R^2^ = 0.504). As per text reading accuracy, a significant main effect of time was revealed (*F*(2, 80) = 6.01; *p* = 0.004) with no age group effects (*F*(1, 54) = 0.69; *p* = 0.409). However, a significant age group*intervention interaction effect emerged (*F*(2, 80) = 3.19; *p* = 0.046). Paired comparison post hoc analyses showed an overall stability in improvement after RT-2 training at T2, only in younger participants (T2 *vs.* T0: t(79) = −3.24, *p* = 0.002; T2 *vs.* T1: t(79) = −1.37, *p* = 0.174), but not in the older ones (T2 *vs.* T0: t(83) = −0.15, *p* = 0.878; T2 vs. T1: t(83) = 1.98, *p* = 0.050) (Figure 2D). The model explained 6.60% of the variance through fixed effects (marginal R^2^ = 0.066), while the full model explained 59.57% of the variance (conditional R^2^ = 0.596).

### 3.5. AIM 2—Educational Impact

Looking at text comprehension performance, a significant improvement was highlighted after RT-2 training (*X*^2^(1) = 6.00, *p* = 0.014), showing that 56.3% of children with below-grade-expected level at T0 performed at grade level at T1. Interestingly, the paired-sample chi-square between T1 and T2 was not significant (*X*^2^(1) = 1.60, *p* = 0.206), suggesting that improvement observed in reading comprehension at T1 remained stable after three months (75.0% of children performing at-grade level at T1 still performed at the same level at T2).

## 4. Discussion

This study aimed to evaluate the stability in reading improvement after a home-based multifaceted training designed to enhance lexical access automatization and visuo-spatial attentional abilities in Italian children with DD. In recent years, computer-based approaches have become increasingly prevalent in DD training, expanding the field to include home-based computerized rehabilitation techniques. Although empirical evidence supporting the efficacy of such interventions in Italian-speaking children have already been reported [12,15,36,40,65], data supporting the stability in reading improvement remains limited [36]. Home-based computerized rehabilitation programs offer the advantage of increasing training intensity and frequency, key factors in skill automatization and treatment efficacy [66,67,68].

The present study agrees with previous evidence supporting improvement in reading proficiency after the RIDInet RT-2 in Italian children with DD [40]. Consistent with prior findings, our results indicate that home-based training can enhance reading speed and accuracy within a relatively short treatment period [15,36]. These findings are consistent with previous studies showing that multi-component interventions targeting lexical, attentional, executive, and phonological processes can improve reading fluency and accuracy [32,33,34,35]. In line with this evidence, the present intervention appeared particularly effective in enhancing lexical access and global reading fluency, as reflected by improvements in single-word and text reading. The recent literature also supports the efficacy of remotely administered interventions in Italian-speaking populations [36,37]. In particular, Lorusso and colleagues [36,37] reported stable gains in reading speed and accuracy following remote lexical–attentional training, while other multi-component programs integrating executive and phonological training showed maintained benefits at 6-month follow-up and positive effects on school achievement [35].

To address the first aim of this study, we compared pre- and post-training scores in reading speed and accuracy among children with DD and examined potential DLD- and age-related differences in treatment efficacy. Overall, regardless of DLD and age, the findings revealed significant enhancements in both speed and accuracy in text and single-word reading, while no significant improvements were observed in single pseudo-words reading [40]. The absence of a significant effect of the RIDInet RT-2 on pseudo-words reading may be explained by the characteristics of transparent orthographies, in which grapheme–phoneme correspondences are highly consistent and decoding skills are typically acquired early and efficiently. As a result, participants may already demonstrate relatively high levels of accuracy and automatization in pseudo-words decoding (see Table 1), limiting the potential for measurable intervention-related gains. This interpretation is consistent with evidence showing that readers of transparent orthographies predominantly rely on small-unit grapheme–phoneme decoding strategies, which are acquired rapidly during reading development [69]. Previous cross-linguistic studies have also shown that pseudo-words decoding accuracy reaches high levels early in transparent languages such as Italian, German, and Finnish, thereby reducing sensitivity to intervention effects in this domain [70]. Consequently, improvements following reading interventions in transparent orthographies are more often observed in fluency or lexical processing measures rather than in basic decoding accuracy.

Overall, our findings align with previous research indicating a specific effect of RIDInet RT-2 on reading speed compared to other training approaches targeting executive functions [10]. Additionally, these results are consistent with evidence showing that different types of interventions (i.e., focused on auditory-based processes, such as rhythm and phonetic training) primarily enhance sublexical processing, including pseudo-word and complex word decoding [19,71]. Furthermore, our findings partially overlap with studies demonstrating significant improvements in word and text reading speed and accuracy following RIDInet RT-2 treatment compared to a waiting-list control, while showing only limited gains in pseudo-word reading [39]. Consistent with previous observations on home-based training, post-treatment efficacy appears comparable across age groups, supporting the notion that treatment should not be restricted to younger children [36]. Given that reading is a dynamic and cumulative process starting from birth and continuing throughout early childhood and adolescence [72,73,74], it is unsurprising that improvements can occur even at later developmental stages [74,75,76].

More importantly, we assessed the stability of improvement in reading speed and accuracy for both text and single-word reading three months after the end of the treatment. Consistent with previous research on home-based interventions, our findings indicate a general stability of pre-post training improvements [36]. Specifically, reading speed remained stable for both single words and text, regardless of comorbidity with DLD and participants’ age. These results can be particularly relevant for treatment planning in clinical practice. Our results show that both children with DD and DLD and those with DD without DLD reached comparable gains in single-word and text reading accuracy and fluency following the intervention, suggesting that the presence of DLD did not attenuate responsiveness to treatment. Although previous data showed that having a diagnosis of DLD impacts reading development and is linked to the risk of DD [46,47,48,49,50,51,52,53,54], other findings did not report any significant difference between the children with DD and those with DD and DLD on reading performance [58,77,78,79]. This could imply that the core mechanisms targeted by the intervention are similarly accessible in individuals with DD regardless of broader language impairments. One possible interpretation is that the intervention primarily acts on domain-specific reading processes that remain relatively independent from higher-order linguistic abilities, including expressive or receptive language skills. Consequently, individuals with co-occurring language difficulties may still benefit from training aimed at improving decoding efficiency and reading fluency to a comparable extent as individuals with isolated DD. However, although longitudinal evidence on DD and DLD reading trajectories is present, limited attention to the effect of DLD comorbidity on reading training efficacy is available. Further research is needed to evaluate how different approaches impact these trajectories both on effectiveness and stability of improvements. As the training used in this study is multi-componential, it may be hypothesized that specific training components differentially benefit distinct clinical sub-groups. Interventions focused on single components such as phonological decoding, rapid automatized naming, reading fluency, or language comprehension may show greater effectiveness depending on the underlying cognitive and linguistic profiles of children with only DD or with DD in comorbidity with DLD. This is particularly relevant if we consider that around 55% of children with DD (recruited from schools or clinics) also meet the criteria for DLD [79].

Moreover, these results can be particularly relevant for treatment planning in older children with DD. Prior studies evaluating interventions targeting early reading precursors, such as auditory processing and phonological awareness, have shown limited efficacy in older children [80]. In contrast, training programs designed to support rapid lexical access and visuo-spatial processing appear beneficial across different age groups [12,15,36]. Notably, reading fluency in transparent orthographies follows a linear developmental trajectory throughout primary school, reaching a plateau in later academic years [76,81,82]. Conversely, while reading accuracy remained stable for single-word reading, its stability in text reading was observed only in younger participants. This aligns with previous findings indicating greater accuracy improvements in younger children compared to older ones [36]. Such results are not surprising in an Italian-speaking sample. Italian orthography is relatively easy to acquire due to its consistent grapheme–phoneme correspondences, compared to other European languages [83]. In transparent orthographies such as Finnish, Italian, Spanish, and German, reading accuracy reaches near-ceiling levels in the first years of schooling, whereas it develops more gradually in opaque languages like French and English. By the end of the first grade, approximately half the Italian-speaking children population can accurately read about 95% of familiar words [81,82,83,84]. Coherently, cross-sectional studies have documented a rapid decline in reading errors during the first three years of primary school [76,82].

Finally, benefits were also observed in reading comprehension both immediately and three months after the end of the training, thus showing further transfer to access the meaning of written text which primarily supports academic performance. These findings are particularly interesting, given that fast and accurate word reading is hypothesized to facilitate reading comprehension because it releases a reader’s cognitive resources (e.g., working memory) to focus on meaning [85,86]. During the process of comprehension, the readers select pieces of information from a text, integrating their knowledge with the information in the text [87]. Reading comprehension is therefore essential for academic success and lifelong learning, supporting the ability to understand, interpret, and critically analyze information [88]. It enables students to process and engage with material effectively, with research showing that strong comprehension skills lead to better academic performance [89].

This study has several limitations that should be considered when interpreting the findings. First, the absence of a control group (e.g., waiting-list condition and/or control treatment) makes it difficult to rule out alternative explanations for the observed effects. Second, the small sample size limits the statistical power and generalizability of the results. Replication studies with larger samples are needed. Third, the relatively short-term follow-up period limits the conclusions on the long-term effects of the RIDInet RT-2 intervention. It is worth noting that very few studies include any follow-up at all, making this a valuable addition to the current literature. Fourth, no specific information was collected regarding the 3-month follow-up period nor individual differences on reading interests and experience during and after training, limiting our ability to characterize variability or account for how these inter-individual factors may have influenced the observed outcomes. Furthermore, no session-by-session data was available for the study, limiting the possibility of assessing individual training parametrization variability and of testing the relationship between training performance/trajectories and outcomes. Fifth, as the primary aim was to evaluate the stability of reading gains following RT-2 training over time rather than to directly compare treatment efficacy, the present study did not include a direct comparison with alternative intervention approaches. Future studies should therefore include different intervention approaches to determine whether some treatments lead to more stable long-term reading outcomes than others. Sixth, RIDINet is not a freely available platform for researchers or clinicians, limiting its diffusion and use. Seventh, since the study was conducted in a transparent language, the findings may not be generalizable to languages with more opaque orthographies. However, given the limited research on Italian-speaking children, this study contributes to filling an important gap in the field. Future research should address the efficacy of training aimed to consolidate rapid lexical access and visuo-spatial processing in opaque languages. Finally, no reliability data for the assessments was available.

## 5. Conclusions

Regardless of differences between training approaches, the current study findings suggest that multi-componential interventions can lead to stable improvements in children with DD, irrespective of DLD and age. Moreover, these results expand upon previous literature showing that benefits are also observed in reading comprehension, which primarily supports academic performance. The current study supports previous research, demonstrating that home-based software can accelerate reading aloud within a few months of training for children with DD in regular orthographies, potentially fostering the automatization of reading processes. These results are promising as home-based exercises are generally well received by children and their families, being perceived as engaging and less demanding than traditional reading remediation activities. Overall, these findings highlight the potential for large-scale, remotely delivered interventions for the most commonly diagnosed learning disorder among school-aged children. Future research should further investigate the specificity of treatment effects and compare training focused on one specific cognitive/sensory function with multi-componential approaches, particularly regarding performance stability.

## Figures and Tables

**Table 1:** Sample descriptive statistics for socio-demographic and clinical variables collected.

	N	Missing	Mean	SD	Minimum	Maximum
Maternal education (years)	52	0	12.29	2.91	8	18
Paternal education (years)	52	0	11.52	2.92	8	18
Global IQ	52	0	98.58	11.3	72	122
Verbal IQ	52	0	105.06	13.65	68	137
Performance IQ	52	0	104.83	12.02	83	132
Single-word reading speed—pre (z-score)	52	0	−3.19	4.1	−23.97	0.53
Single-word reading accuracy—pre (z-score)	52	0	−2.04	2.24	−10.60	1
Single pseudo-word reading speed—pre (z-score)	51 *	1	−1.62	2.33	−11.79	1.2
Single pseudo-word reading accuracy—pre (z-score)	51 *	1	−1.59	1.87	−6.33	1
Text reading speed—pre (z-score)	52	0	−2.03	1.06	−7.40	−0.22
Text reading accuracy—pre (z-score)	52	0	−0.97	0.59	−1.65	0.67

*Note.* IQ—Intelligence Quotient at the Wechsler Intelligence Scale for Children—Fourth Edition (mean = 100; standard deviation = 15); pre—pre-training assessment. * For 1 of the participants, the single pseudo-word reading test performance was not available due to scarce assessment adherence from the child.

## Data Availability

Data will be available upon reasonable request to the corresponding author.

## Supplementary Materials

The following supporting information can be downloaded at: https://www.mdpi.com/article/10.3390/brainsci16060636/s1, Figure S1. Significant pre-post differences (z-scores for outcome variables presenting a significant increase in performance: single-word reading speed (A), accuracy (B) and text reading speed (C) and accuracy (D). Figure S2. Significant pre-post differences and follow-up stability (z-scores) for outcome variables presenting a significant increase in performance: single-word reading speed (A), accuracy (B) and text reading speed (C) and accuracy (D). Table S1. Stability in reading improvement after a home-based multi-componential training for children with developmental dyslexia. Table S2. Descriptives and independent sample *t*-test results comparing demographic and clinical measures between groups with and without 3-month follow-up. Supplementary Analysis. Stability Models with Global IQ covariate (and interaction).
