# Supplementary material for: Stability in Reading Improvement After Home-Based Multi-Componential Training for Children with Developmental Dyslexia
Source: Brain Sci. 2026 Jun 14;16(6):636. doi: 10.3390/brainsci16060636 (PMC13296689; doi:10.3390/brainsci16060636)

Supplementary Materials S1 - Stability in reading improvement after a home-based multi-componential training for children with developmental dyslexia

Table S1 – Descriptives clinical measures at pre-post training and 3-month follow-up

	**T0 (pre)**					**T1 (post)**					**T2 (follow-up**				
**Performance (z-scores)**	**N**	**Mean**	**SD**	**Min**	**Max**	**N**	**Mean**	**SD**	**Min**	**Max**	**N**	**Mean**	**SD**	**Min**	**Max**
**Word reading speed**	52	-3.19	4.10	-23.97	0.53	52	-1.73	3.35	-16.96	1.62	26	-1.83	4.48	-22.41	1.21
**Word reading accuracy**	52	-2.04	2.24	-10.60	1.00	52	-1.15	1.97	-10.70	1.00	26	-0.92	1.49	-5.00	1.00
**Pseudo-word reading speed**	51	-1.62	2.33	-11.79	1.20	52	-1.19	1.97	-8.76	1.22	26	-2.08	4.99	-25.23	1.37
**Pseudo-word reading accuracy**	52	-1.56	1.87	-6.33	1.00	52	-1.25	1.53	-5.00	1.25	26	-1.11	1.48	-4.25	0.80
**Text reading speed**	52	-2.03	1.06	-7.40	-0.22	52	-1.32	1.06	-3.88	2.26	27	-1.62	0.95	-3.15	0.40
**Text reading accuracy**	52	-0.98	0.59	-1.65	0.67	52	-0.69	0.68	-1.65	0.67	27	-0.64	0.72	-1.65	0.67

At first assessment 33 out of 52 (63.5%) experienced below grade average reading comprehension performances. At post training assessment 20 out of 52 (39.2%) and similarly at follow-up 11 out of 27 (40.7%) experienced below grade average reading comprehension performances.

Table S2 – Descriptives and independent sample t-test results comparing demographic and clinical measures between groups with and without 3-month follow-up

	NO follow-up		Follow-up			
	**Mean**	**SD**	**Mean**	**SD**	***t(df)***	***p***
Age at pre-training assessment (years)	9.00	1.44	8.30	1.32	1.83 (50)	.073
Maternal education (years)	12.00	2.92	12.50	2.94	-0.59 (50)	.559
Paternal education (years)	10.80	2.67	12.10	3.05	-1.64 (50)	.107
Global IQ	98.76	10.95	98.41	11.82	0.11 (50)	.912
Single word reading speed – pre (z-scores) *	-3.50	5.26	-2.90	2.67	-0.52 (35)	.607
Single word reading acc – pre (z-scores)	-2.62	2.57	-1.50	1.76	-1.86 (50)	.069
Single pseudo-word reading speed – pre (z-scores)	-1.89	2.63	-1.36	2.03	-0.81 (39)	.424
Single pseudo-word reading acc – pre (z-scores)	-1.51	1.93	-1.20	1.59	-0.60 (44)	.550
Text reading speed – pre (z-scores)	-2.08	1.36	-1.99	0.69	-0.31 (50)	.761
Text reading acc – pre (z-scores)	-1.08	0.53	-0.88	0.64	-1.20 (50)	.235
Days of treatment (number in 12 weeks)	69.68	24.15	79.48	35.53	-1.15 (50)	.254

* Levene's test is significant ($p < .05$), suggesting a violation of the assumption of equal variances – Welsh t-test was used

Note: IQ – Intelligence Quotient (mean = 100; standard deviation = 15); pre – pre-training assessment

Supplementary Materials S2 - Stability in reading improvement after a home-based multi-componential training for children with developmental dyslexia

Figure S1 – Significant pre-post differences (z-scores)

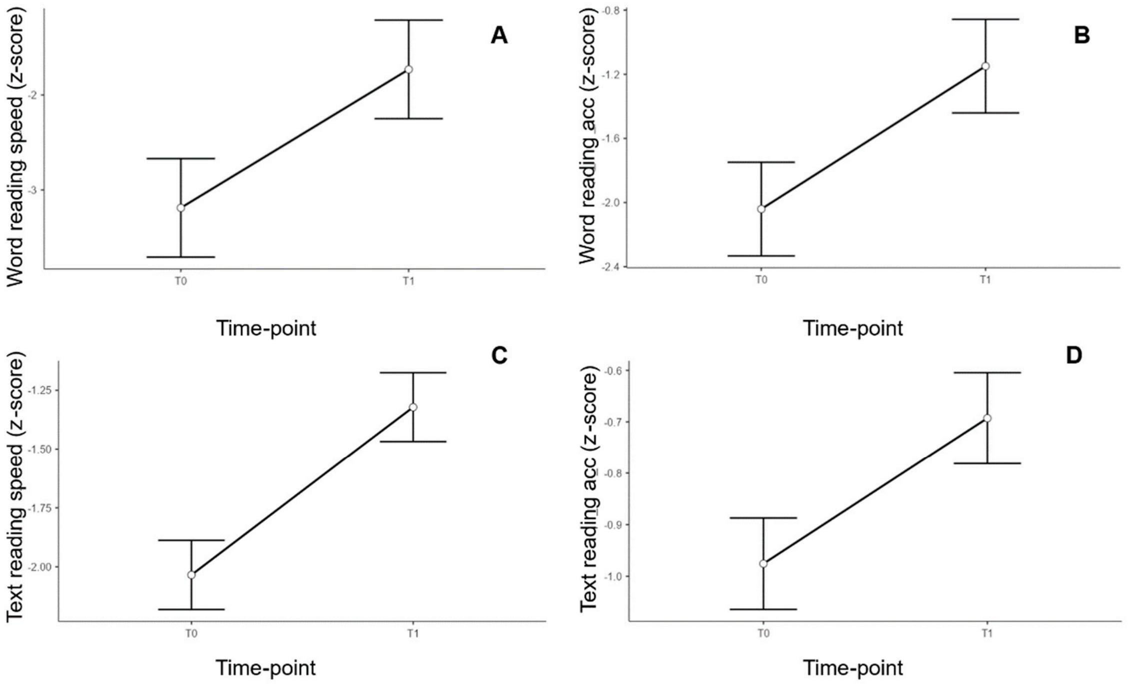

Figure S2 – Significant pre-post differences and follow-up stability (z-scores)

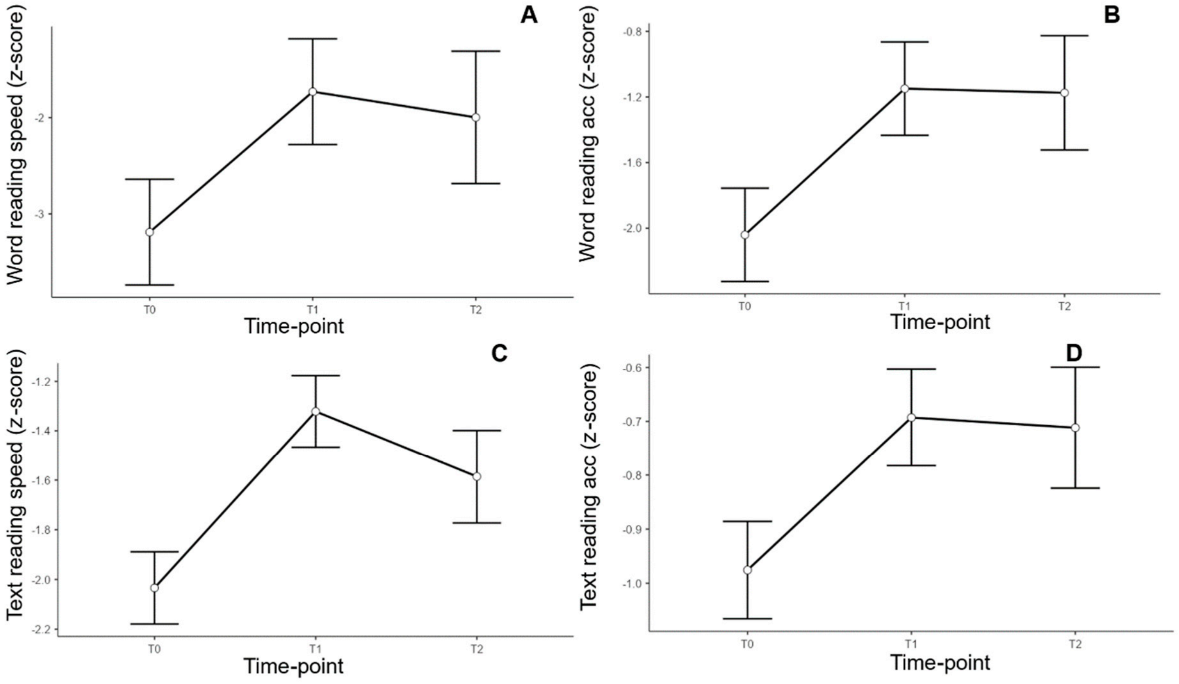

Stability Models with Global IQ covariate (and interaction)

Mixed model analyses showed significant effects on Global IQ in predicting reading performance in speed components (word and text) with positive estimates, Global IQ predicted z scores closer to normative mean in reading speed. No main effects of Global IQ emerged in accuracy. Finally, no significant interaction emerged between Time-point and Global IQ suggesting that intellectual functioning did not moderate training results. Importantly, in all models the main effect of Time-point remained significant even controlling for Global IQ effects or Time-point x Global IQ interactions.

Mixed Model - Word reading speed 2

Mixed Model - Word reading accuracy 4

Mixed Model - Text reading speed 6

Mixed Model - Text reading accuracy 8

Mixed Model - Word reading speed

Word reading speed (z) ~ 1 + Global IQ + Time-point + Global IQ:Time-point + (1 | ID)

Model Results

Model Fit

Type	R^2	df	LRT X^2	p
Conditional	0.620	6	44.961	<.001
Marginal	0.109	5	17.159	0.004

Fixed Effects Omnibus Tests

	F	df	df (res)	p
**Global IQ**	6.37	1	50.2	0.015
**Time-point**	4.67	2	76.1	0.012
Global IQ ✻ Time-point	1.56	2	75.7	0.217

Parameter Estimates (Fixed coefficients)

				95% Confidence Intervals				
**Names**	**Effect**	**Estimate**	**SE**	**Lower**	**Upper**	**df**	**t**	**p**
(Intercept)	(Intercept)	-2.3063	0.4727	-3.2420	-1.3705	51.7	-4.879	<.001
Global IQ	Global IQ	0.1058	0.0419	0.0228	0.1888	50.2	2.524	0.015
Time-point1	T1 - T0	1.4590	0.4937	0.4818	2.4363	72.6	2.956	0.004
Time-point2	T2 - T0	1.1897	0.6403	-0.0779	2.4572	78.5	1.858	0.067
Global IQ ✻ Time-point1	Global IQ ✻ (T1 - T0)	-0.0101	0.0441	-0.0974	0.0773	72.6	-0.229	0.820
Global IQ ✻ Time-point2	Global IQ ✻ (T2 - T0)	0.0831	0.0550	-0.0257	0.1919	77.8	1.512	0.135

Random Components

Groups	Name	Variance	SD	ICC
**ID**	(Intercept)	8.55	2.92	0.574
**Residual**		6.34	2.52	

Note. Number of Obs: 130 , Number of groups: ID 52

Post Hoc comparison: Time-point

**Comparison**							
**Time-point**	**vs**	**Time-point**	**Difference**	**SE**	**t**	**df**	**p**
T0	-	T1	-1.459	0.494	-2.955	72.6	0.004
T0	-	T2	-1.190	0.640	-1.859	78.5	0.067
T1	-	T2	0.269	0.640	0.420	78.5	0.676

Results Plots

Time-point ✻ Global IQ

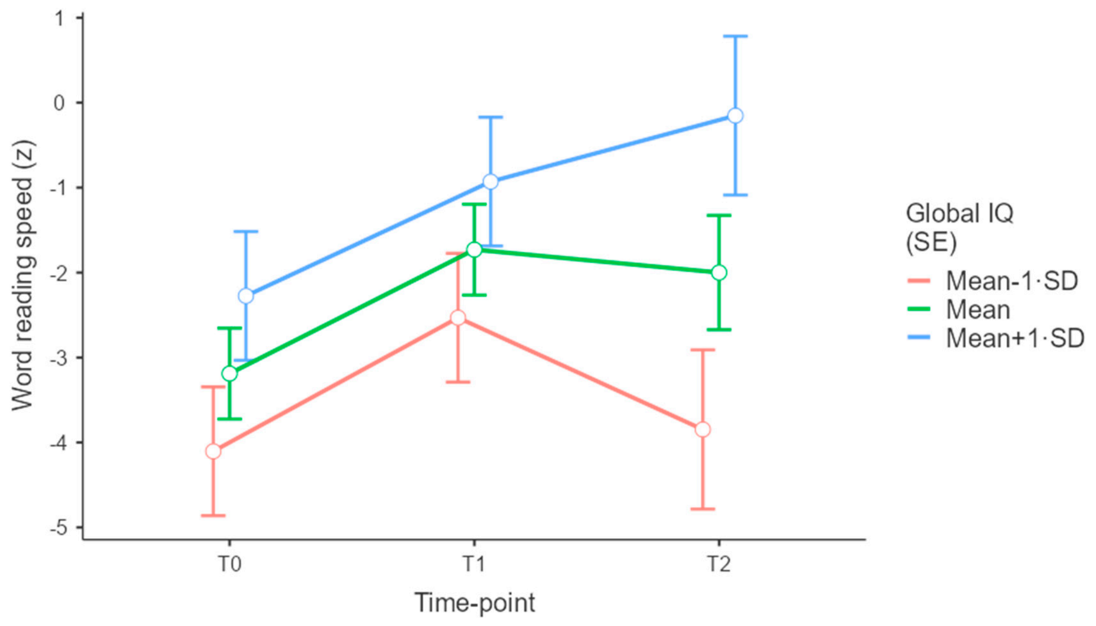

Mixed Model - Word reading accuracy

Word reading acc (z) ~ 1 + Time-point + Global IQ + Global IQ:Time-point + (1 | ID)

Model Results

Model Fit

Type	R^2	df	LRT X^2	p
Conditional	0.658	6	47.868	<.001
Marginal	0.053	5	16.462	0.006

Fixed Effects Omnibus Tests

	F	df	df (res)	p
**Time-point**	7.763	2	75.0	<.001
**Global IQ**	0.501	1	49.7	0.482
**Time-point ✻ Global IQ**	0.543	2	74.7	0.583

Parameter Estimates (Fixed coefficients)

				95% Confidence Intervals				
**Names**	**Effect**	**Estimate**	**SE**	**Lower**	**Upper**	**df**	**t**	**p**
(Intercept)	(Intercept)	-1.45455	0.2580	-1.9654	-0.9438	50.9	-5.634	<.001
Time-point1	T1 - T0	0.89135	0.2430	0.4103	1.3724	72.2	3.668	<.001
Time-point2	T2 - T0	0.86614	0.3164	0.2399	1.4924	77.0	2.738	0.008
Global IQ	Global IQ	0.01621	0.0229	-0.0291	0.0616	49.7	0.708	0.482
Time-point1 ✻ Global IQ	(T1 - T0) ✻ Global IQ	0.02228	0.0217	-0.0207	0.0653	72.2	1.026	0.308
Time-point2 ✻ Global IQ	(T2 - T0) ✻ Global IQ	0.00662	0.0271	-0.0471	0.0603	76.4	0.244	0.808

Random Components

Groups	Name	Varianc e	SD	ICC
**ID**	(Intercept)	2.71	1.65	0.639
**Residual**		1.54	1.24	

Note. Number of Obs: 130 , Number of groups: ID 52

Post Hoc Tests

Post Hoc comparison: Time-point

Comparison							
**Time-point**	**vs**	**Time-point**	**Difference**	**SE**	**t**	**df**	**p**
T0	-	T1	-0.8915	0.243	-3.6686	72.2	<.001
T0	-	T2	-0.8662	0.316	-2.7379	77.0	0.008
T1	-	T2	0.0253	0.316	0.0800	77.0	0.936

Results Plots

Time-point ✻ Global IQ

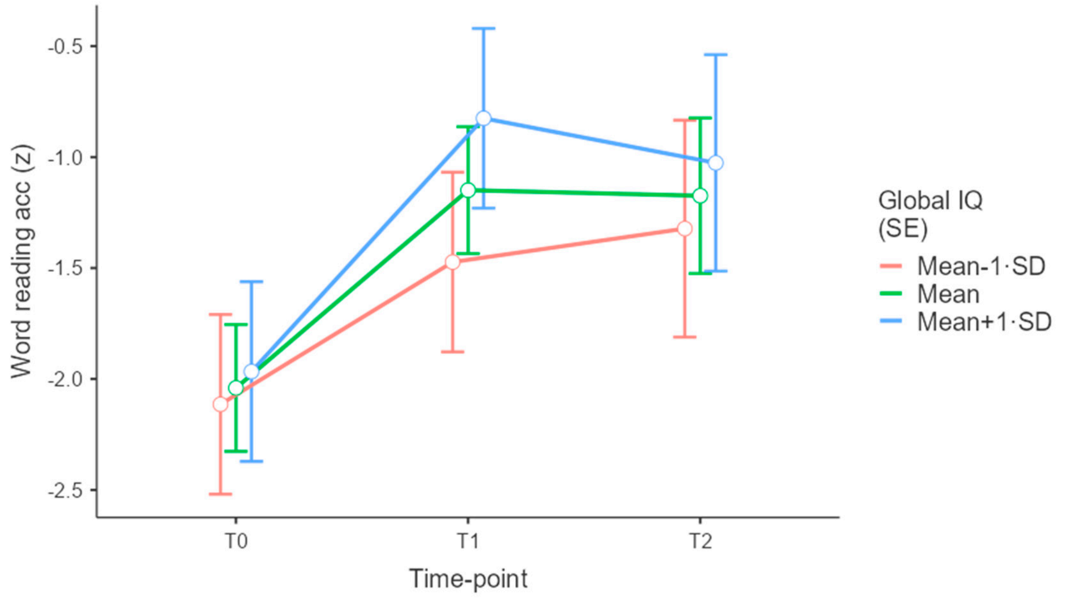

Mixed Model - Text reading speed

Text reading speed (z) ~ 1 + Global IQ + Time-point + Global IQ:Time-point + (1 | ID)

Model Results

Model Fit

Type	R^2	df	LRT X^2	p
Conditional	0.506	6	36.650	<.001
Marginal	0.148	5	26.016	<.001

Fixed Effects Omnibus Tests

	F	df	df (res)	p
**Global IQ**	5.6812	1	50.1	0.021
**Time-point**	11.0483	2	78.4	<.001
**Global IQ ✻ Time-point**	0.0523	2	77.9	0.949

Parameter Estimates (Fixed coefficients)

				95% Confidence Intervals				
**Names**	**Effect**	**Estimate**	**SE**	**Lower**	**Upper**	**df**	**t**	**p**
(Intercept)	(Intercept)	-1.64706	0.1177	-1.88006	-1.4141	51.5	-13.9922	<.001
Global IQ	Global IQ	0.02488	0.0104	0.00422	0.0455	50.1	2.3835	0.021
Time-point1	T1 - T0	0.71212	0.1524	0.41039	1.0138	73.3	4.6718	<.001
Time-point2	T2 - T0	0.44826	0.1928	0.06657	0.8299	82.0	2.3247	0.023
Global IQ ✻ Time-point1	Global IQ ✻ (T1 - T0)	-0.00392	0.0136	-0.03089	0.0230	73.3	-0.2878	0.774
Global IQ ✻ Time-point2	Global IQ ✻ (T2 - T0)	2.99e-4	0.0168	-0.03288	0.0335	81.1	0.0178	0.986

Random Components

Groups	Name	Variance	SD	ICC
**ID**	(Intercept)	0.439	0.662	0.421
**Residual**		0.604	0.777	

Note. Number of Obs: 131 , Number of groups: ID 52

Post Hoc comparison: Time-point

**Comparison**							
**Time-point**	**vs**	**Time-point**	**Difference**	**SE**	**t**	**df**	**p**
T0	-	T1	-0.712	0.152	-4.67	73.3	<.001
T0	-	T2	-0.448	0.193	-2.32	82.0	0.023
T1	-	T2	0.264	0.193	1.37	82.0	0.175

Results Plots

Time-point ✻ Global IQ

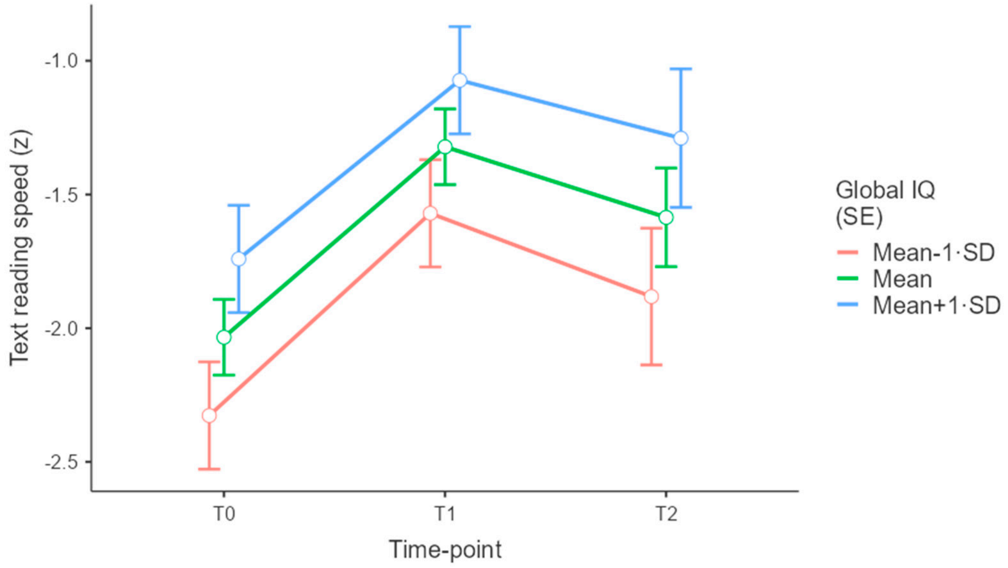

Mixed Model - Text reading accuracy

Text reading acc (z) ~ 1 + Time-point + Global IQ + Global IQ:Time-point + (1 | ID)

Model Results

Model Fit

Type	R^2	df	LRT X^2	p
Conditional	0.570	6	42.922	<.001
Marginal	0.047	5	13.266	0.021

Fixed Effects Omnibus Tests

	F	df	df (res)	p
**Time-point**	6.1273	2	80.2	0.003
**Global IQ**	0.0345	1	53.4	0.853
**Time-point ✻ Global IQ**	0.5765	2	79.9	0.564

Parameter Estimates (Fixed coefficients)

				95% Confidence Intervals				
**Names**	**Effect**	**Estimate**	**SE**	**Lower**	**Upper**	**df**	**t**	**p**
(Intercept)	(Intercept)	-0.79382	0.07898	-0.9501	-0.6375	54.5	-10.051	<.001
Time-point1	T1 - T0	0.28269	0.08598	0.1125	0.4529	76.7	3.288	0.002
Time-point2	T2 - T0	0.26258	0.10967	0.0455	0.4797	82.7	2.394	0.019
Global IQ	Global IQ	0.00130	0.00702	-0.0126	0.0152	53.4	0.186	0.853
Time-point1 ✻ Global IQ	(T1 - T0) ✻ Global IQ	0.00472	0.00768	-0.0105	0.0199	76.7	0.614	0.541
Time-point2 ✻ Global IQ	(T2 - T0) ✻ Global IQ	-0.00532	0.00953	-0.0242	0.0135	82.1	-0.558	0.578

Random Components

Groups	Name	Variance	SD	ICC
**ID**	(Intercept)	0.233	0.483	0.549
**Residual**		0.192	0.438	

Note. Number of Obs: 131 , Number of groups: ID 52

Post Hoc Tests

Post Hoc comparison: Time-point

Comparison							
**Time-point**	**vs**	**Time-point**	**Difference**	**SE**	**t**	**df**	**p**
T0	-	T1	-0.2825	0.0860	-3.286	76.7	0.002
T0	-	T2	-0.2628	0.1097	-2.396	82.7	0.019
T1	-	T2	0.0198	0.1097	0.180	82.7	0.857

Results Plots

Time-point ✻ Global IQ

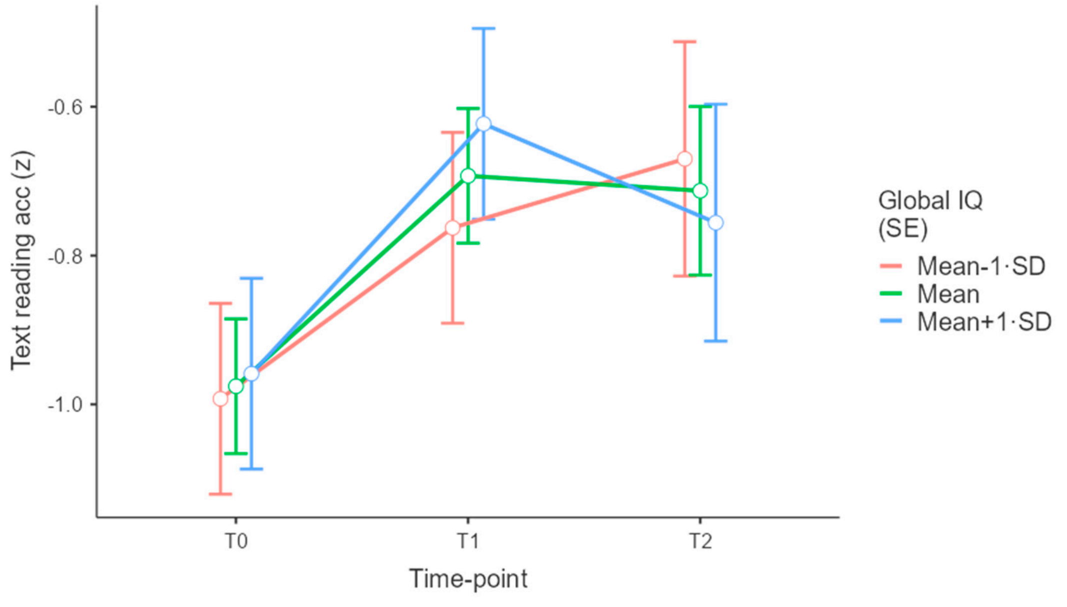

Supplement: Supplementary file 1 [file brainsci-16-00636-s001.zip › brainsci-4225365-supplementary.pdf]
